# Experimental and simulation investigation on suppressing thermal runaway in battery pack

**DOI:** 10.1038/s41598-024-62408-1

**Published:** 2024-06-03

**Authors:** Zibo Ye, Xingfeng Fu

**Affiliations:** 1 School of Automobile and Transportation Engineering, Guangdong Polytechnic Normal University, Guangzhou, 510665 China; 2 GAC Aion New Energy Automobile Co., Ltd., Guangzhou, 511434 China

**Keywords:** Thermal runaway, Battery management system, Simulation modeling, Suppression design, Energy science and technology, Engineering

## Abstract

In order to address the issue of suppressing thermal runaway (TR) in power battery, a thermal generation model for power batteries was established and then modified based on experimental data. On the basis of simulation calculations, a scheme was designed to suppress thermal runaway of the battery module and battery pack, and samples were produced for testing. The results of the test and simulation calculations were very consistent, confirming the accuracy of the simulation calculation model. The results of thermal runaway test also demonstrate that the measures designed to suppress thermal runaway are effective and meet the design requirements.

## Introduction

With the rapid development of electric vehicles (EVs), lithium-ion power battery systems as power sources have received more and more attention. With the continuous increase in battery specific energy, the range of EVs has approached or exceeded that of fuel vehicles. Consequently, the safety of power batteries has also attracted increasing attention. Given the substantial energy stored in the power battery system of EVs, any occurrence of thermal runaway or thermal diffusion can lead to severe fire and explosion incidents, posing a significant threat to the safety of both vehicles and drivers^1,2^. Therefore, effective measures must be taken to prevent and suppress the occurrence and effects of thermal diffusion in power batteries. This is crucial for safeguarding the safety of EVs and drivers.

The thermal diffusion experiment of power batteries has become one of the most important projects in the safety testing of power batteries. It is a mandatory project for regulatory inspections, an essential aspect of power battery system design, and a key evaluation criterion for assessing the quality of such designs. At present, the prevention and suppression technology of thermal diffusion in power batteries has become one of the most important development focuses in power battery system design, attracting the attention of domestic and foreign researchers^3,4^.

From the data of fire accidents caused by thermal runaway in lithium-ion batteries, it can be seen that the consequences of thermal runaway in individual lithium-ion batteries are limited. However, the propagation behavior of thermal runaway can often exacerbate the consequences of thermal runaway and lead to more serious accidents. The heat transfer process in the battery pack is the fundamental reason for the propagation of thermal runaway within the battery module. Therefore, theoretically speaking, if the generation of heat during the thermal runaway process of the battery can be suppressed, and even if the heat between the batteries is transferred, it can effectively reduce the damage effect of thermal runaway in lithium-ion batteries. This is the prevention and suppression technology of thermal runaway in lithium-ion batteries. From the current research results of domestic and foreign scholars, the suppression methods for thermal runaway of lithium-ion batteries mainly include insulation and expanding heat dissipation capacity. At present, some scholars have found that changing the material formula inside lithium-ion batteries, selecting noncombustible battery materials to make batteries, or adding some flame retardant materials to the structure of batteries can significantly reduce the generation of internal heat during the thermal runaway process of batteries, or significantly reduce the battery temperature during the thermal runaway process of batteries. These have a significant effect on reducing the risk of thermal runaway in lithium-ion batteries. In addition, adding epoxy resin plates of different thicknesses between battery packs can block the thermal conduction and radiation capabilities between batteries, prolong the thermal conduction process between different batteries, and effectively reduce the thermal runaway damage caused by lithium-ion batteries^5–7^.

This article will conduct research on the prevention and suppression of thermal runaway in lithium-ion power batteries from these two aspects. The work of this manuscript can provide useful references for the design of a safety management system for electric vehicle battery packs.

### Factors influencing the results of battery thermal runaway

The main factors that cause TR in electric vehicles are external battery abuse and internal short circuits resulting from the internal battery. External abuse includes mechanical, electrical, and thermal abuse. Internal short circuits are primarily caused by battery damage resulting from puncturing the battery separator or exceeding the permitted charging and discharging capacity of the battery. Generally, mechanical, electrochemical, and thermal conditions will be combined, and the TR of the battery is the result of multiple factors^5–7^.

Mechanical abuse mainly refers to battery deformation caused by external forces, commonly observed in typical situations such as car collisions and battery compression. The focus of researchers is on mechanical excursion, which investigates whether the deformation of a battery caused by external force will result in an internal short circuit. The concern is whether this short circuit will lead to thermal runaway and ultimately result in the explosion of the battery. Collision squeezing is a common form of mechanical abuse that is typically studied at three levels: component, cell, and module or pack^8^. The mechanical model for extrusion of battery cells is usually based on analyzing the mechanical properties of the materials that make up the cells^9^. A novel aluminum honeycomb design module is proposed to mitigate TR propagation by offering improved mechanical performance in both quasi-static state compression and high-speed impact crash scenarios^10^. In fact, the battery underwent significant deformation before the onset of the internal short circuit. Therefore, predicting internal short circuits caused by mechanical abuse at the cell level has become one of the research hotspots. The failure behavior and damage tolerance of a battery pack, based on a detailed model including enclosure and jellyroll components, are being investigated^11^. Lithium-ion batteries are closely related to the protective ability of the entire package against impacts in various directions when subjected to mechanical abuse at model level.

**Figure 1:**
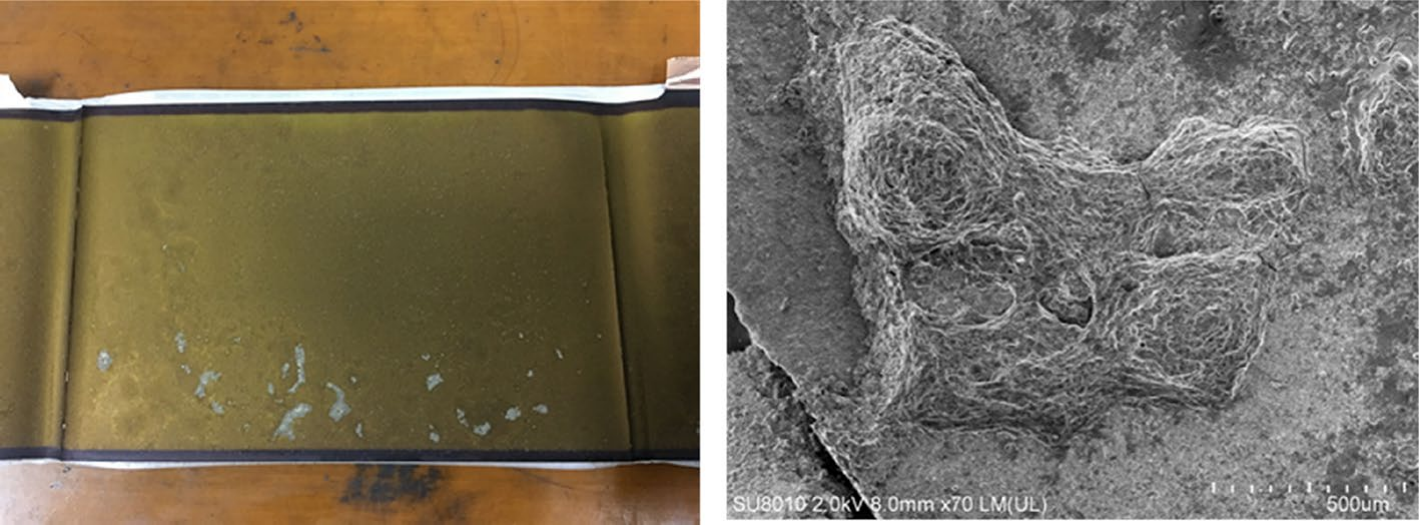
Morphology of lithium evolution and deposition of lithium metal on the negative electrode of power batteries under overcharging.

Statistics indicate that overcharging is a prevalent issue that leads to electricity abuse, causing thermal runaway in power batteries^12,13^. This issue has led to numerous safety accidents associated with TR in the practical application of EVs. The direct cause of overcharging is the failure of the battery management system (BMS) protection measures to promptly cut off the charging current when the power battery is fully charged, resulting in charging beyond the capacity of the power battery. Overcharging typically results in heating and gas generation, primarily stemming from Ohmic heat and side reaction heat^14,15^. Research has found that the heat generated by batteries is directly proportional to the charging current, indicating that ohmic heat is one of the main reasons for heat generation during the charging process. Our previous work^16^ demonstrated that overcharging can cause significant damage to power batteries. As illustrated in Fig. 1, due to excessive lithium embedding in the negative electrode, lithium dendrites can form on the surface of the negative electrode. Excessive detachment of lithium in the positive electrode can result in structural collapse. The generation of heat in the cells accelerates the release of oxygen, leading to the decomposition of the electrolyte and the production of a large amount of gas. After a sharp increase in internal pressure, the power battery may ignite or explode^17–19^. Sun et al.^20^ have studied the risk factors associated with external short circuits (ESCs) at the module level. During the breaking process of the weak link, an arc re-breakdown will be generated as the battery voltage rises. The higher the battery current, the greater the energy of the arc, leading to the immediate vaporization of the surrounding metal and causing significant damage to the battery. In the design of the module, it is crucial to incorporate external short circuit protection devices such as weak links and fuse to effectively mitigate external short circuits of the battery.

Local temperature spikes in the battery pack are a common form of thermal abuse condition^21^. Nonconforming contact interfaces between the electrode brackets and collector bars, as well as non-uniform pressure distribution, will lead to higher electrical contact resistance and power loss. Tab overheating in pouch-type lithium-ion batteries, which is considered to be more prone to thermal runaway, has a faster propagation speed in an open environment. In the thermal runaway propagation experiments of large-format lithium-ion cells, overheating was selected as the initial trigger mechanism for thermal runaway^22–24^. Dynamic mechanical loads caused by shocks, drops, or vibrations may result in mandrel displacement or contact loss, contributing to an increase in the ohmic resistance of the batteries. The damaged connections caused by vibrations, resulting from the detachment of the welded joint of the bent conductor, may fail completely. This can lead to arc generation and, consequently, thermal runaway^25^.

Figure 1 shows the disassembly results of a 94Ah lithium-ion power battery developed by our research group after more than 200 tests of 6C rate charging and discharging. From the left figure in Fig. 1, it can be seen that while adjusting the formula to improve the charging and discharging rate of lithium-ion power batteries, white lithium precipitated on the negative electrode inside the battery, and bubble marks and black spots appeared on the accessories. The SEM film structure in the right figure was also damaged, and irreversible damage had already occurred inside the battery. The battery damage was quite obvious. In the subsequent battery thermal runaway experiment, the battery caught fire violently, and the internal characteristics displayed were consistent with the judgment of the experimental results. Therefore, it is necessary to find ways to redesign the battery to avoid lithium deposition and SEM film damage during use. After these two designs meet the requirements, the thermal diffusion characteristics parameters of the power battery will be measured through EV-ARC experiments to design targeted measures to prevent thermal runaway and meet the requirements of thermal diffusion testing of the power battery.

In addition to the three types of external abuse mentioned above, the battery's own defects may also cause internal short circuits. When the internal separator of the battery is damaged and the positive and negative poles come into contact with each other, an internal short circuit occurs. Internal short circuit is one of the prominent characteristics of thermal runaway^26^. After an internal short circuit occurs, the electric energy and electrochemical energy stored in the electrode plate will be released violently, generating a large amount of heat in a short time, leading to runaway heating.

## Research on suppression designs of power battery thermal runaway

Once a battery cell experiences TR, it may propagate to the neighboring batteries, accompanied by the generation of combustible mixed gases, ultimately leading to serious fire and explosion events^27,28^. An unexpected failure mode is that the spread of gas and flame can cause thermal runaway diffusion. The reason why the first pathway is expected is mainly because it is relatively easy to block heat transfer. However, the unexpected nature of the second pathway is primarily attributed to the uncertainty surrounding gas and flame diffusion. These two failure pathways can be suppressed through the following methods including heat insulation devices, current interrupting devices, safety vents, and fire extinguishments^29–32^.

**Figure 2:**
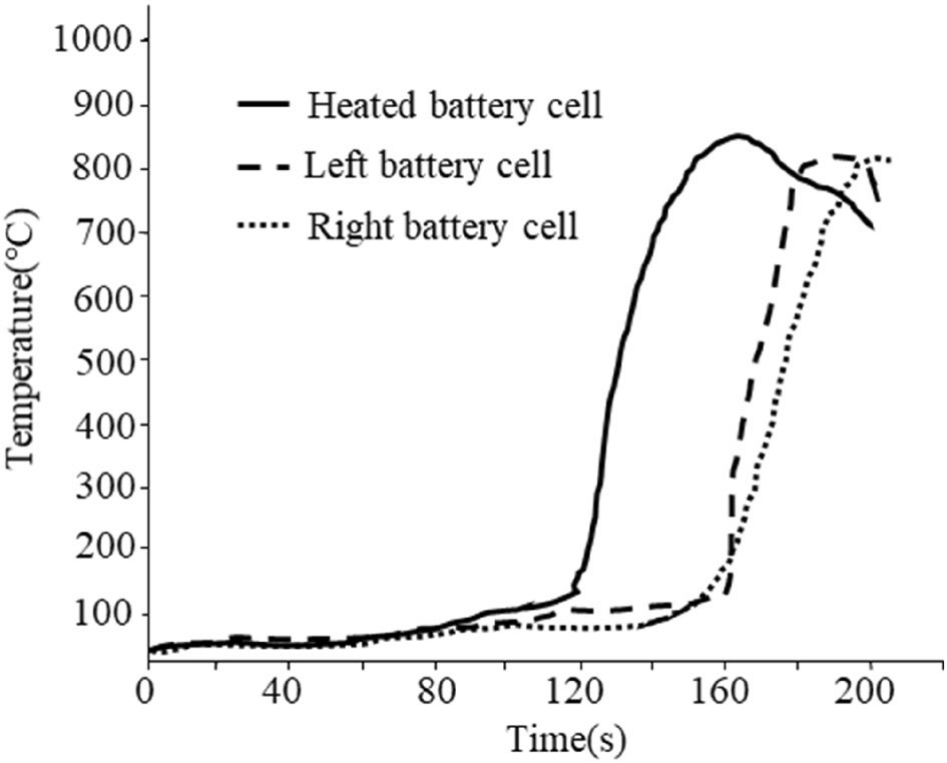
Experiment result diagram of Thermal runaway of battery center temperature.

To prevent thermal runaway diffusion in power battery systems, researchers aim to minimize the propagation of failure following the first exploded battery^33^. Figure 2 displays the TR test process diagram of a prismatic power battery. It can be seen from the figure that under the influence of the high temperature of the heating wire, the TR of the target battery triggered a chain reaction of TR in the two batteries surrounding the module. In a short time, the two affected batteries caught fire first, and the maximum temperature during the runaway of the battery could reach over 800 °C, far exceeding the battery’s TR control threshold. This led to internal damage to the battery and the ejection of high-temperature substances. Upon the expulsion of polar materials within the battery, the battery shell ruptured, leading to the ejection of most internal materials. This process resulted in the direct release of heat energy to the battery’s surroundings, consequently lowering the internal temperature of the battery. Under the pressure of the gas inside the battery, the direction of the sprayed material is relatively random and highly uncertain. During the battery TR test, the temperature sensor inside the battery module was damaged. As a result, the battery temperature information was no longer available after the first 200 s of the test.

It can be seen from the test results in Fig. 2 that there is a need to find a method to control the path and energy of external heat diffusion following a single-cell TR. In the initial module structure design, after the TR of a battery is triggered, a significant amount of heat and combustible materials inside the battery are expelled onto the adjacent battery. This leads to the successive TR of adjacent batteries, as confirmed by the disassembly analysis of the cell. It is very important to study the associated failure methods of batteries during the battery runaway process.

Figure 2 is the Thermal runaway test process diagram of a square power battery. The internal materials and formula of this 94Ah battery have been redesigned. After 500 consecutive charge and discharge tests at a rate of 6C, it was found that the negative electrode did not precipitate lithium when disassembling the battery, and the metallographic structure of the SEM film remains intact. Assemble the redesigned battery into a module for thermal runaway testing. It can be seen from the figure that under the influence of the high temperature of the heating wire, the thermal runaway of the target battery caused a chain reaction of Thermal runaway of the two batteries around the module. In a short time, the two affected batteries caught fire first, and the maximum runaway temperature of the battery could reach more than 800 °C, which has far exceeded the Thermal runaway control threshold of the battery, This caused internal damage to the battery and the ejection of high-temperature substances. With the ejection of polar materials inside the battery, the battery shell ruptured, and the vast majority of internal materials were ejected, causing heat energy to be directly released to the outside of the battery, resulting in a decrease in the internal temperature of the battery. Under the pressure of the gas inside the battery, the direction of the sprayed material inside the battery is relatively random and has great uncertainty. During the battery Thermal runaway test, the temperature sensor inside the battery module was damaged, so the battery temperature date recorded of Battery Thermal runaway test disappeared after about 200 s.

It can be seen from the test results in the figure that it is necessary to find a way to restrain the path and energy of external heat diffusion after a single cell Thermal runaway. In the initial module structure design, after the Thermal runaway battery is ignited, a large amount of heat and combustible materials inside the battery are sprayed onto the adjacent battery, resulting in the successive Thermal runaway of adjacent batteries, which is confirmed from the disassembly analysis of the cell. It is very important to study the associated failure methods of batteries during the process of battery runaway.

## Heat generation simulation model of power battery

### Simulation calculation model for battery heat generation

The actual heat production of the battery is complex. The simulation calculation process for battery TR is similar to the calculation model for battery heat generation. Therefore, some assumptions about the physical properties of the battery itself should be made in the simulation calculation.

- The specific heat capacity and thermal conductivity of various materials inside the battery are not affected by changes in ambient temperature or state of charge.
- The medium of various materials in the battery is evenly distributed, and the thermal and physical parameters remain unchanged. For example, the thermal conductivity coefficient of the same material is equal in the same direction.
- During the charging and discharging of lithium-ion batteries, the current density is evenly distributed, and the rate of heat production is consistent at different temperatures.

Through the above assumptions, the energy conservation equation for unsteady heat transfer is derived.

1 $$\rho {c}_{p}\frac{\partial T}{\partial t}={\lambda }_{x}\frac{{\partial }^{2}T}{{\partial x}^{2}}+{\lambda }_{xy}\frac{{\partial }^{2}T}{{\partial y}^{2}}+{\lambda }_{zx}\frac{{\partial }^{2}T}{{\partial z}^{2}}+q$$

${\rho }_{k}{c}_{p,k}\frac{\partial T}{\partial t}$ refers to the increase of the thermal mechanical energy of the battery unit within a unit time,$\nabla \cdot \left({\lambda }_{k}\nabla T\right)$ refers to the heat added to the cells inside the battery due to convective heat transfer by the fluid around the battery, q is the rate of heat production per unit volume of a lithium-ion battery, ρk is refers to the average density of the cell, c_p_, k is refers to the average specific heat capacity of lithium-ion battery cells, λ_k_ is refers to the thermal conductivity of the lithium-ion battery unit, T is thermal, t is time, $\rho$ is the average density of the material inside a lithium-ion battery, $q$ is heat production rate per unit volume of a lithium-ion battery, ${\lambda }_{x}$,${\lambda }_{y}$ and ${\lambda }_{zx}$ are thermal conductivity of a lithium-ion battery in three-dimensional orthogonal directions.

Many parameters in the chemical reaction kinetics formula that require calibration must be measured using various sets of experimental equipment. The common test method is constant-temperature scanning calorimetry, and the common experimental equipment is differential scanning calorimetry (DSC). DSC can scan the sample at a constant rate of temperature increase. By comparing the difference between the heat absorbed or released by the sample and the reference material at a constant temperature rise rate, the heat release or absorption of the sample can be determined.

If a single battery within the battery pack is used, the formula for the temperature $\text{T}$ of the battery as a function of time $\text{t}$ is as follows:

2 $$\text{T}\left(t\right)=T\left(0\right)+\underset{0}{\overset{t}{\int }}\frac{dT\left(\tau \right)}{d\tau }d\tau $$

The temperature rise rate $\frac{dT\left(t\right)}{dt}$ is determined by the net heat generating power $\text{Q}\left(t\right) is$ inside the battery, where M is the battery mass and the Specific heat capacity of the battery is ${C}_{p}=1100J.{kg}^{-1}{K}^{-1}$.

3 $$Q\left(t\right)={Q}_{chem}\left(t\right)+{Q}_{e}\left(t\right)-{Q}_{h}\left(t\right)$$

4 $${Q}_{chem}\left(t\right)={Q}_{SEI}\left(t\right)+{Q}_{anode}\left(t\right)+{Q}_{sep}\left(t\right)+{Q}_{e}\left(t\right)+{Q}_{cath}\left(t\right)$$

5 $${Q}_{cath}\left(t\right)={Q}_{caht1}\left(t\right)+{Q}_{caht2}\left(t\right)$$

${Q}_{chem}\left(t\right)$ is chemical reaction heat generation power, ${Q}_{SEI}\left(t\right)$ is solid electrolyte interphase (SEI) membrane Chemical decomposition, ${Q}_{anode}\left(t\right)$ is the heat generation power of lithium metal embedded inside the negative electrode when it reacts with the electrolyte without the protection of the SEI film, ${Q}_{sep}\left(t\right)$ is the heat absorption power of the diaphragm during melting, ${Q}_{e}\left(t\right)$ is the exothermic power of the overall Chemical decomposition of electrolyte solution, ${Q}_{cath}\left(t\right)$ is thermal power generation during decomposition of ternary cathode materials. Since the reaction of ternary positive electrode has two exothermic peaks ${Q}_{caht1}\left(t\right)$ and ${Q}_{caht2}\left(t\right)$ , the two Exothermic reaction are only equal to ${Q}_{cath}\left(t\right)$.

6 $${Q}_{e}\left(t\right)=\frac{1}{\Delta t}\left(\Delta {H}_{e}-\underset{0}{\overset{t}{\int }}{Q}_{e}\left(\tau \right)d\tau \right)$$

The calculation of Qe can be obtained from the following equation, $\Delta {H}_{e}$ represents the total electrical energy possessed by the battery when an internal short circuit occurs; ∆t represents the average time of electrical energy release. The adiabatic Thermal runaway model can simulate the dynamic characteristics of each chemical reaction, and it is calculated in the model。The maximum temperature of Thermal runaway should be consistent with the experimental results. Its main principle is the Conservation of energy, $\Delta \text{t}$ represents the total heat energy released in the process of Thermal runaway; M represents the quality of the battery; ${C}_{p}$ represents the Specific heat capacity of the battery; $\Delta T$ represents the maximum temperature rise of battery Thermal runaway, according to formula (2–6), ∆ T = T3-T1; ${\Delta \text{H}}_{chem}$ represents the total amount of Chemical energy converted into heat energy in the process of Thermal runaway; ${\Delta \text{H}}_{e}$ represents the total amount of electric energy converted into heat energy in the process of Thermal runaway.

7 $$\Delta \text{H}=\text{M}\cdot {C}_{p}\cdot \Delta T={\Delta \text{H}}_{chem}+{\Delta \text{H}}_{e}$$

8 $${\Delta \text{H}}_{chem}=\sum_{x}\left({C}_{x,0}\cdot \Delta {H}_{x}\cdot {m}_{x}\right)$$

${\Delta \text{H}}_{chem}$ is determined by the properties of the material itself and can be calculated from the data in the formula, where ${\Delta \text{H}}_{chem}$ is approximately 2.87 × 10^5^ J. According to the energy conservation equation and the experimentally measured ∆ T, set ${\Delta \text{H}}_{e}$ approximately 3.17 × 10^5^ J.

### Simulation calculation model of battery thermal runaway

Based on the above analysis results, it is essential to maintain an adiabatic testing environment to accurately test and obtain battery samples. The rate of temperature rise dT_S_/dt of the product under adiabatic conditions should aim to eliminate the influence of sensor measurement errors. Therefore, the adiabatic rate calorimeter (EV-ARC) should be calibrated before the experiment. During the calibration process, the heat dissipation environment of the calorimeter should closely resemble the environment during the actual TR test. The heat dissipation of the calorimeter chamber needs to consider not only the heat dissipation from its outer wall to the environment of the experimental site but also the impact caused by the heat absorption of the battery sample.

The key points of using EV-ARC to conduct thermal insulation of thermal runaway tests for large-capacity power batteries include:

1. Thermal insulation for environmental control;
2. Accurate detection of the heat release rate dT_S_/dt.

In order to address the two points mentioned above, the following tasks should be carried out during the experiment:

1. Before conducting the formal experiment on thermal insulation of thermal runaway, mitigate the impact of sensor measurement errors by implementing a calibration scheme involving the replacement of sensors with equal heat capacity and mechanical clamping.
2. During the calibration process, try to recreate the internal environment of the calorimetry chamber, including large-capacity battery samples. This will help achieve more accurate heat dissipation compensation calibration results.
3. During formal testing, pay attention to using the sensor’s mechanical clamping scheme to ensure that the sensor is tightly attached to the surface of the battery.

It should also be noted that the temperature distribution inside the large-capacity power battery is uneven. When TR occurs, the temperature distribution becomes highly uneven. In order to accurately evaluate the total energy released by the large-capacity power battery during thermal runaway, it is essential to measure the internal temperature of the battery. Additionally, analyzing the changes in internal temperature differences during the experiment is crucial.

**Figure 3:**
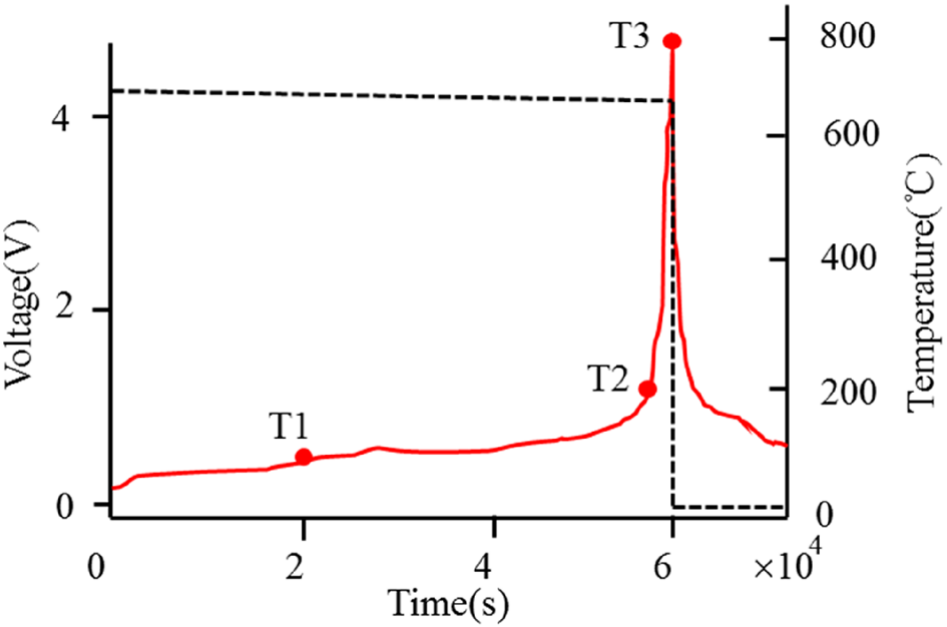
Accelerating Rate Calorimeter (ARC) data of sample battery.

Figure 3 shows the results obtained from the ARC experiment. It can be seen that T1 represents the initial temperature of battery self-heating, while T2 represents the threshold temperature for thermal runaway, which is approximately 200 °C. At this point, the rate of change of temperature (dT/dt) changes sharply, and T3 represents the highest temperature reached during battery thermal runaway. This temperature value is very high and can serve as the reference temperature set by the battery system protection device. The results of this test are consistent with the previous simulation results of the battery.

It can be seen from the figure that when the temperature reaches about 60 °C, the solid dielectric facial mask (SEI film) on the negative electrode surface begins to decompose. At this point, the negative electrode of the battery loses SEI film protection. The lithium embedded inside the negative electrode then comes into contact with the electrolyte, triggering a reaction that releases heat and generates a new SEI film. The loss of lithium in the negative electrode of the battery leads to a rise in the negative voltage. Under high-temperature conditions, metal ions within the positive electrode of the battery dissolve in the electrolyte. This leads to the depletion of active substances in the positive electrode, consequently reducing the voltage of the positive electrode. Due to the voltage of lithium-ion power batteries being the difference between the positive and negative voltage, the battery’s voltage also decreases.

The theoretical policy model of battery thermal runaway will be based on experiments, and key parameters obtained through EV-ARC, especially the information and occurrence time of temperature points T1, T2, and T3, will be used as parameters to construct simulation parameter curves, which will be imported into the battery heat generation calculation model to construct the battery thermal runaway calculation model. Through multiple experiments and simulation calculations, the fitting parameters were corrected to obtain the thermal runaway simulation calculation model for this battery. The simulation calculation model is the theoretical basis for us to analyze and calculate whether the preventive measures for battery thermal runaway are effective.

**Figure 4:**
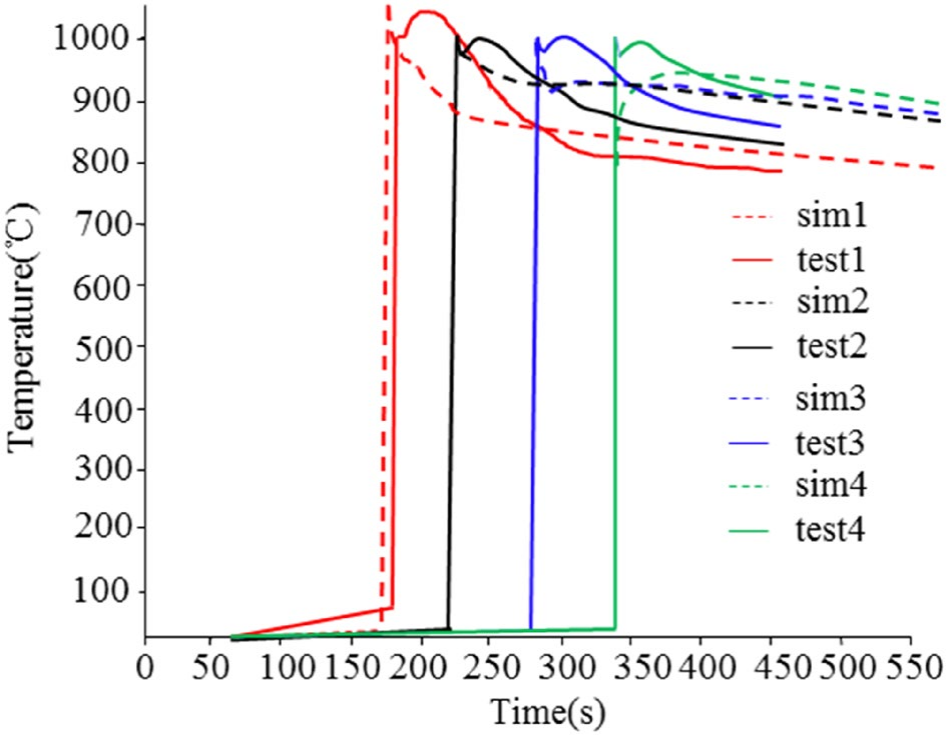
Experiment and simulation diagram of thermal runaway of battery center temperature.

Figure 4 shows the comparison between the simulation calculation results and the experimental results of multiple battery center temperature points during the battery Thermal runaway test. These temperature sensors are embedded inside the battery to be measured. The internal center temperature of the battery is measured. See Table 1 for the simulation calculation values and measurement results inside the battery. It can be seen from the data in Fig. 4 and Table 1 that the simulation calculation results are very close to the measured results of the battery, with an accuracy of more than 90%, Therefore, the simulation calculation model of battery heat generation should be used to analyze the process of Thermal runaway of batteries.

**Table 1:** Simulation and test results of battery inner temperature.

	Cell1	Cell2	Cell3	Cell4
Simulation (°C)	1005.2	1005.1	1004.8	995.7
Test (°C)	1055.0	998.8	957.6	940.3
Accuracy (%)	95.3	99.4	95.3	94.4

## System design for suppressing battery thermal runaway

There are numerous strategies to enhance the suppression of thermal diffusion in power batteries, and targeted design can be implemented from three perspectives: battery cells, battery modules, and battery systems. At the cell level, without compromising the fundamental performance of the battery, incorporating flame retardants into the battery electrolyte and opting for SEI films that are more heat-resistant are both effective strategies for minimizing the severity of thermal runaway damage. Due to limitations in the length of this article, detailed measures to prevent thermal diffusion at the cell level will not be discussed. Our research focus of this paper is the suppression of TR at the battery module and pack levels.

### Battery module thermal runaway suppression design

**Figure 5:**
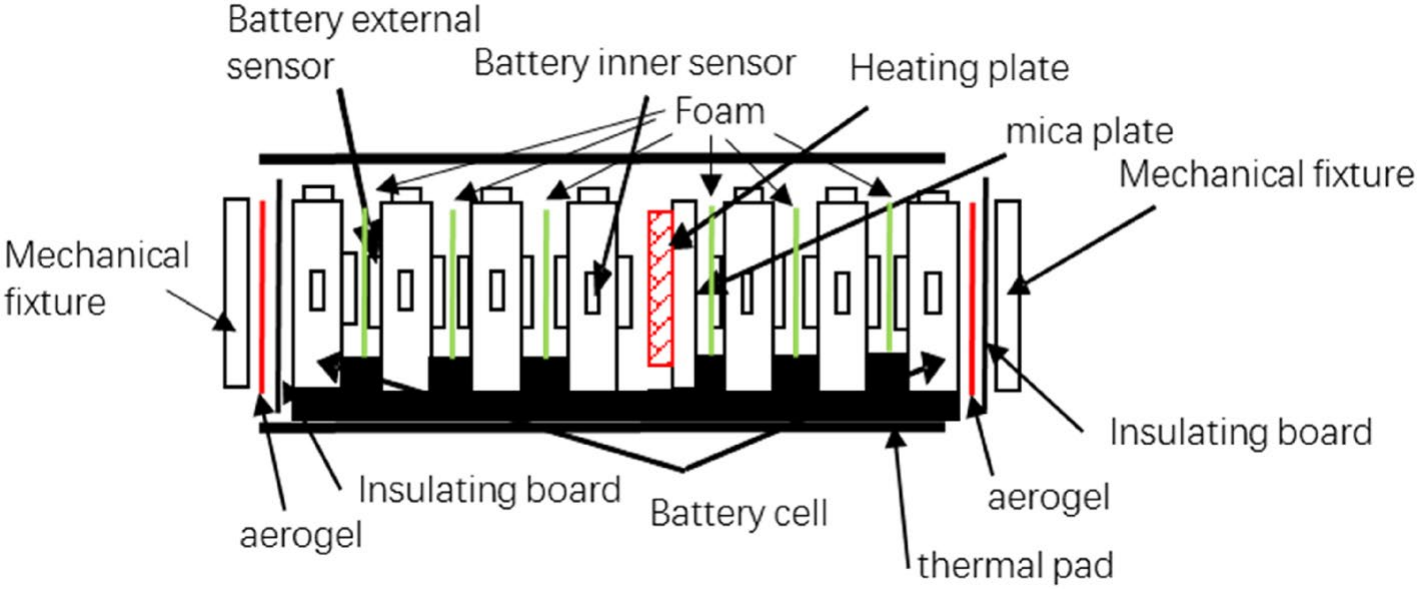
Design of thermal runaway suppression for battery modules.

For the thermal runaway test on the target module, the first step involves designing a heating device that matches the appearance and size specifications of the actual module within the battery pack. This device will replace a module in the center of the battery pack. Temperature sensors will be embedded both inside and outside the heating device, as well as within the electric core of the module. The power supply for the heating plate will be connected to the external trigger power supply outside the battery pack using a wire. Additionally, the heating plate will be installed along with the thermal conductive mica sheet. The design of the heating plate in the middle of the module is shown in Fig. 5.

Since the design size of the heating plate in the module is essentially the same as the size of the battery cell in the original module, the introduction of the heating plate will not have a significant impact on the structure of the battery module under test. This, in turn, will not affect the deviation of the TR experiment from the TR effect of the actual battery module structure. Add a 1.0 mm thick aerogel layer between the end plate and the PC (Polycarbonate)plate. The thermal conductivity of aerogel is extremely low, approximately 0.025 W/(m K), which is only 12.5% of that of the PC plate. Added thermal resistance between the battery cells at both ends of the module and the module end plate to effectively block heat conduction on the end plate side.

To enhance observation of the heating target module on the heating plate and prevent accidental triggering of thermal runaway in the surrounding modules. Therefore, on the other side of the heating plate, there is also a thermal insulation mica sheet. During the test, the heating plate only heats the target battery in the module, without affecting the surrounding batteries, to maintain the integrity of the experimental results.

### Design for suppressing thermal runaway in battery systems

**Figure 6:**
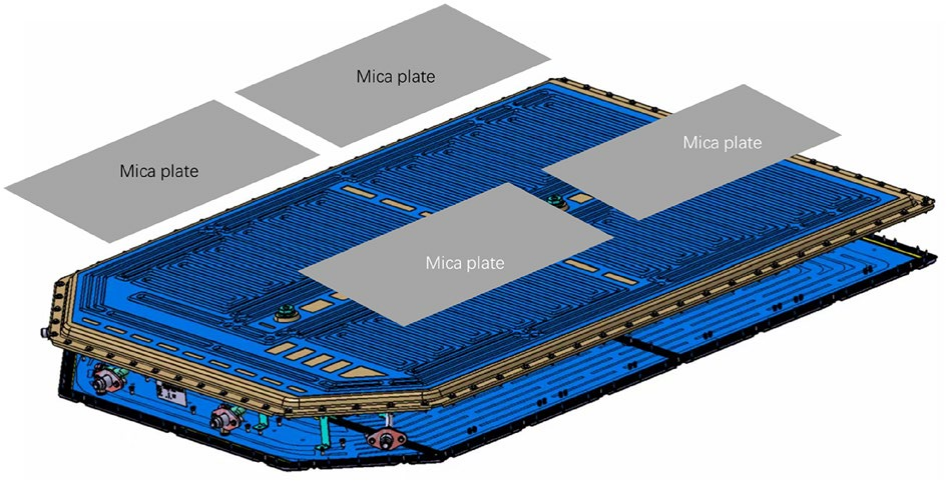
Design of thermal runaway suppression for battery packs.

In the tested module, a temperature sensor is installed inside the power battery when it is completed in the factory, and fluorescent substances are added to the electrolyte. This allows for the observation of temperature changes in the heating plate and battery shell during the experiment, as well as the measurement of temperature changes inside the battery. These measures provide valuable data support for designing a scheme to suppress TR of the battery. At the same time, by adding fluorescent substances to the electrolyte, it is possible to observe and measure electrolyte eruption during TR of the power battery after the experiment. This provides valuable insights for enhancing the strategy to suppress thermal runaway in power battery packs. As shown in Fig. 6.

The function of the mica plate is to isolate the flames and high-temperature gases sprayed from the battery during a fire from damaging the upper shell of the battery, dispersing heat and preventing heat from concentrating on a metal shell that is prone to burning through the battery. In addition, the number of vent valves on the box has been increased from 2 to 4, and the vent pressure limit of the vent valves has been lowered, allowing the gas generated during the battery thermal runaway experiment to be released from the outside of the battery pack more quickly, preventing high-pressure gas accumulation inside the battery pack, effectively reducing the risk of battery pack explosion.

### Design of software for suppressing thermal runaway in battery systems

According to the changes in battery temperature, internal resistance, voltage, and insulation resistance values obtained during the experiment, the criteria for determining battery thermal runaway in the battery management system have been adjusted. For instance, the BMS evaluates the rate of cell voltage reduction, the temperature of the battery casing, and the temperature fluctuations on the mica chip for ten consecutive cycles. This process helps detect thermal runaway faults at an earlier stage and accurately report them, thereby reducing the likelihood of false alarms and failures to report thermal runaway faults.

**Figure 7:**
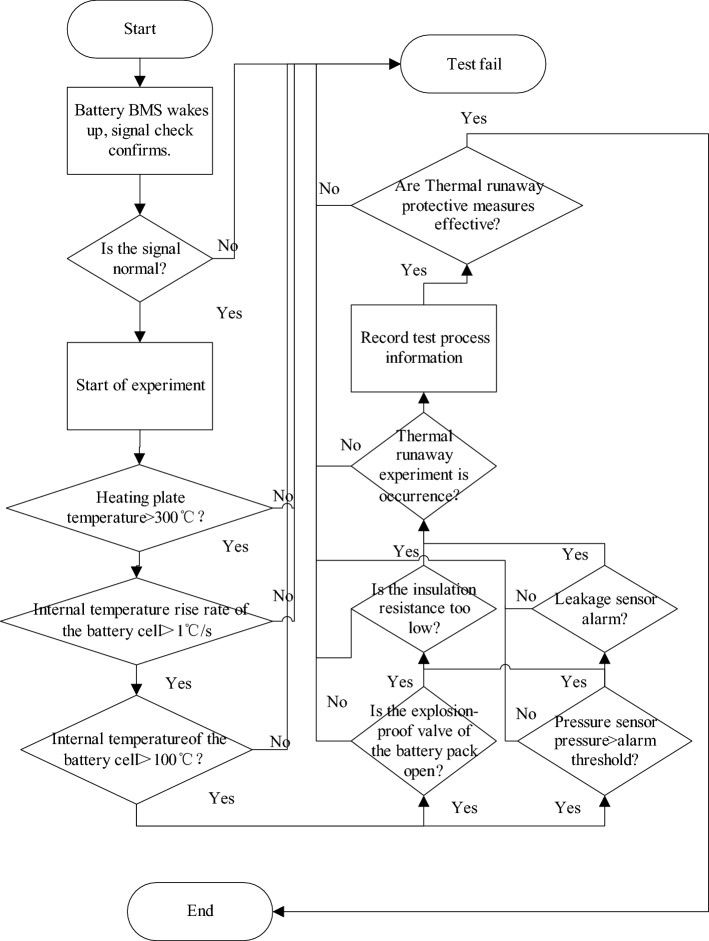
Thermal runaway judgment process.

As shown in Fig. 7, the BMS needs to repeatedly confirm the thermal runaway signal. It compares the measured internal and external temperature of the battery, coolant temperature, flow information, battery insulation, explosion-proof valve pressure, and other relevant data. By making a final judgment through repeated comparisons, the BMS aims to prevent any misjudgment of thermal runaway.

## Results of the experiment

**Figure 8:**
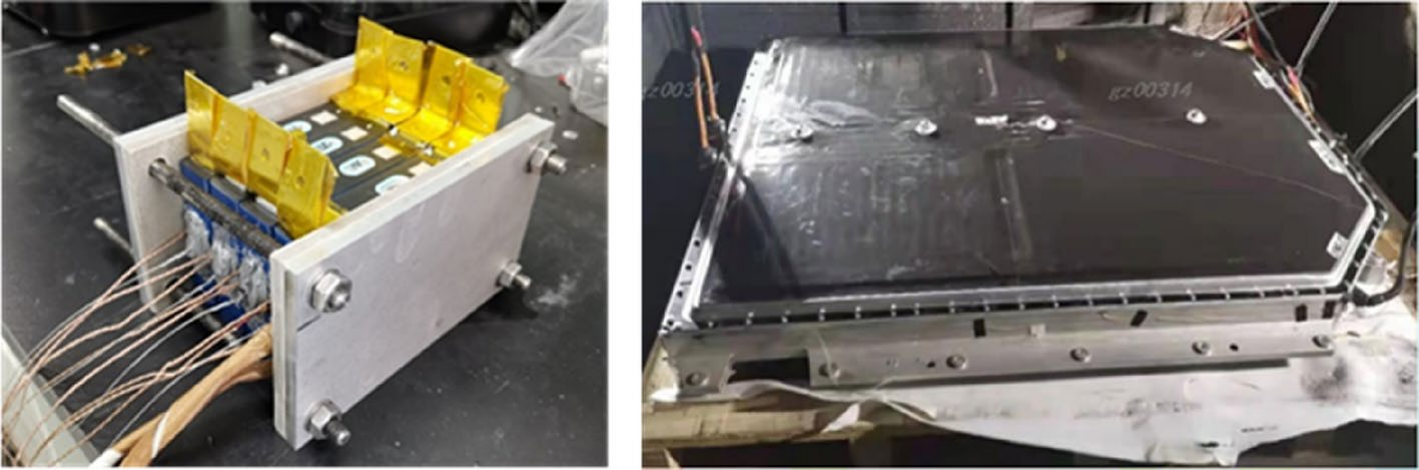
The battery module and battery pack for thermal runaway test.

The thermal runaway test of the entire battery pack will be conducted on the test bench. The left photo in Fig. 8 shows the modified thermal runaway trigger module of the power battery. In this module, measures to suppress thermal runaway have been added in accordance with the previous design requirements. Thermocouples have been embedded inside the cell, and temperature sensors are arranged on the surface of and at the edge of the cell. At the same time, it will monitor the individual voltage and insulation resistance of the battery. On the right of Fig. 8 is a photo showing the battery pack being placed into the explosion-proof temperature chamber for a thermal runaway test.

The battery pack being tested will be fully charged in advance to simulate the most severe thermal runaway scenario, ensuring that the battery has maximum energy during testing. The specific method involves charging to 4.2 V with constant current and voltage, and calibrating it to 100% state of charge (SOC). All temperature sensors are monitored and recorded in real-time during the thermal runaway experiment.

**Figure 9:**
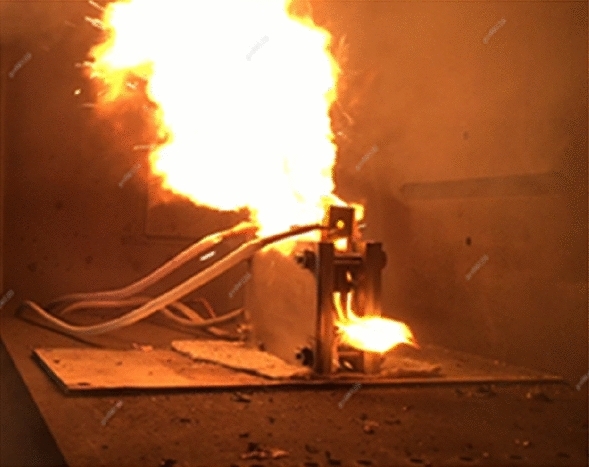
The battery module thermal runaway test.

**Figure 10:**
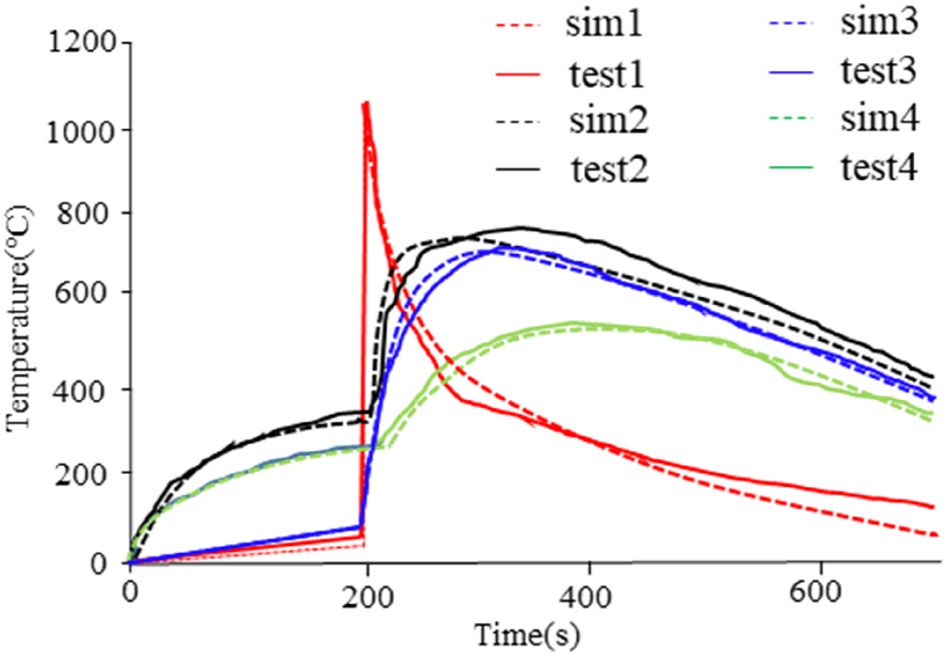
Experiment and simulation diagram of thermal runaway of battery module.

Figure 9 shows a photo of the battery module thermal runaway test conducted in isolation. It can be seen from the photo that during the entire thermal runaway test process, high-temperature substances were ejected from the ignited battery, and the measured internal temperature of the battery cell exceeded 1000 °C. Under such high temperatures, the SEI film and other substances have been decomposed and damaged. Within the same module, the temperature of the three surrounding batteries also rapidly increased, but no explosion or fire was detected. The measured temperature values are shown in Fig. 10.

From the experimental results in Fig. 10, it can be seen that the thermal runaway suppression design inside the module was effective. The heat generated by the thermal runaway module was transferred to the cooling plate through the thermally conductive pad, and the cooling plate dissipated a portion of the heat. At the same time, the fireproof materials between different electric cores in the same module also play a role in flame retardancy. This prevents the surrounding electric cores from being ignited by the thermal runaway battery. The experimental results are consistent with the simulation results, which also demonstrate the accuracy of the simulation calculation model. At the same time, the protection measures inside the module have achieved the design goal by safeguarding the surrounding batteries.

**Figure 11:**
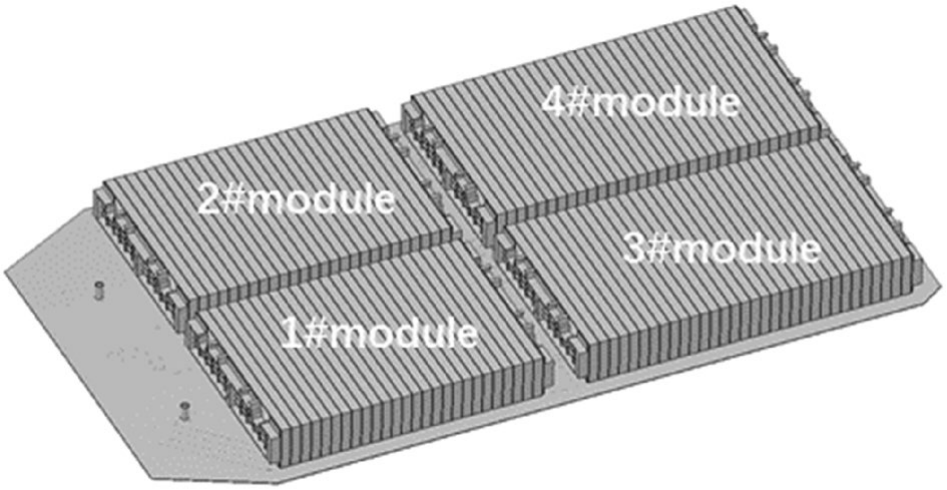
The module layout of battery pack.

After completing the thermal runaway test at the battery module level, install the designed thermal runaway trigger module into the power battery box for a thermal runaway test of the entire battery package. This battery pack consists of four large internal modules. One module serves as the thermal runaway trigger, while the temperature signals of the other three modules are collected by the temperature sensors embedded within each module. The temperature sensor settings are entirely consistent with the structure of the battery pack in the mass production version. The distribution diagram of the modules inside the battery pack is shown in Fig. 11.

**Figure 12:**
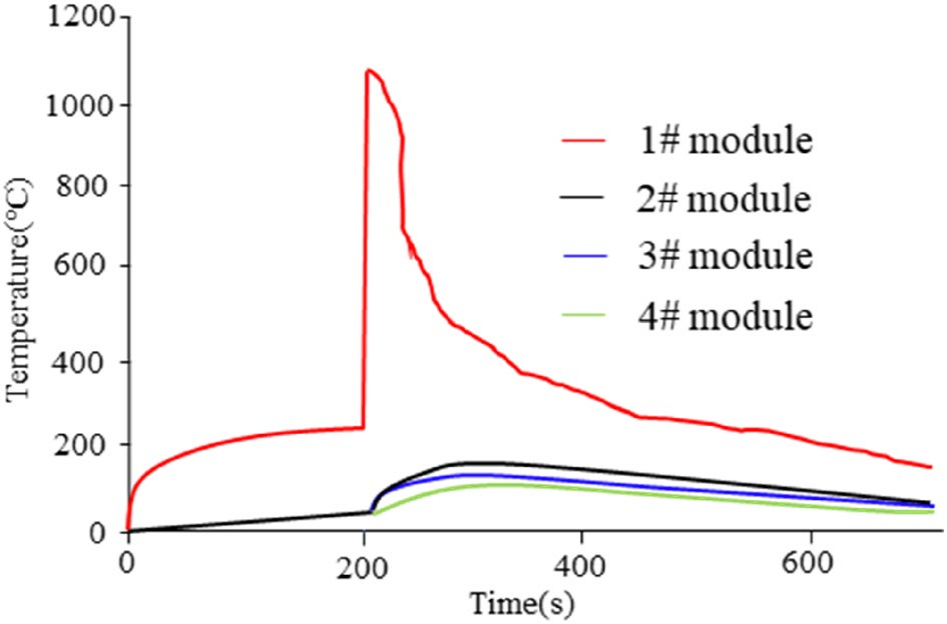
EV battery pack thermal runaway test.

Figure 12 displays the measured temperature data obtained during the TR experiment for 4 modules within the entire battery pack. It can be seen from the figure that the triggering process of the thermal runaway module met the design expectations. The TR was triggered quickly, 200 s later, with the temperature rising sharply. The temperature of the three adjacent modules increased gradually, but all remained below 200 °C. According to the previous analysis, it can be considered that as long as the temperature remains below 200 °C, batteries will not cause dangerous accidents such as explosions and fires. During the entire testing process, once the TR was initiated, one battery module released a significant amount of heat and gas, resulting in the bulging and deformation of the upper cover of the battery pack. However, because the four explosion-proof valves designed on the battery pack box were opened in time to release the air pressure, the entire battery box remained intact.

**Figure 13:**
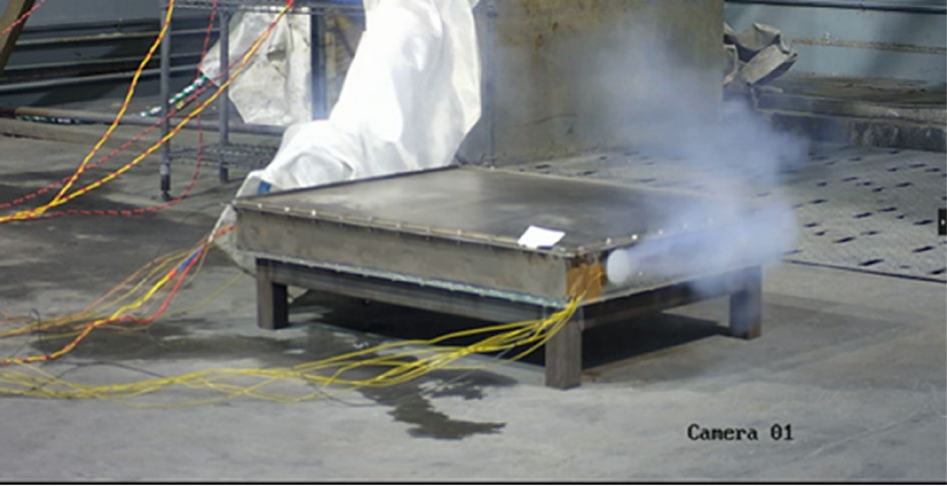
The test picture of battery system thermal runaway.

Figure 13 shows the test photos of the battery thermal runaway experiment. From the experimental video and pictures recorded by a high-speed camera, it can be seen that after adding measures to prevent thermal runaway, the thermal runaway battery module was heated until it caught fire and burned, and the battery box remained intact without being burned through. As shown in the figure, after the battery catches fire and burns, the high-temperature gas and flame emitted by the 1# battery module were isolated by the mica plate on the battery box cover. As a result, the steel plate on the battery pack cover was not burned through and remained intact. This effectively avoids the risk of explosion of the battery box. The entire testing process meets the criteria for battery pack thermal runaway, demonstrating that the implemented protective measures are effective and align with the design specifications.

## Conclusions

This paper studies the TR protection technology of power batteries. By establishing a thermal simulation model of a power battery and incorporating experimental data to adjust the battery’s thermal model, it is possible to accurately simulate the TR process of a power battery. On this basis, targeted protection design is implemented to minimize thermal damage to the battery during thermal runaway. Only samples are produced for actural vehicle verification. The main achievements of this article are as follows:

1. The thermal diffusion simulation calculation model of the power battery has been established, and the key parameters of the model have been adjusted based on the experimental data. The model can accurately simulate the TR process of the battery.
2. Targeted design modifications have been implemented in the power battery to suppress thermal runaway. Additionally, specific adjustments have been made to the battery module and pack designs. The experimental results demonstrate that the designed protective measures meet the design requirements and have a positive protective effect.
3. The research results of this manuscript show that aerogel can effectively cut off the direct heat transfer of different battery cells, and prevent the surrounding battery cells from being ignited when the heat of a battery cell is out of control; Mica board can disperse the heat emitted by the battery, preventing heat from accumulating at a certain point and causing the battery casing to burn through. The simulation and experimental results demonstrate that these preventive measures for thermal runaway have a significant effect on preventing battery thermal runaway.

The research results of this article have been applied to the design of battery packs for mass-produced EV vehicles, achieving ideal results in comprehensive consideration of design schemes and cost control. It can provide valuable reference for similar battery pack designs to prevent thermal runaway.

## Data Availability

The date that support the findings of this study are available from the corresponding author upon reasonable request.
